# VEGF-C expression attributes the risk for lymphatic metastases to ovarian cancer patients

**DOI:** 10.18632/oncotarget.17978

**Published:** 2017-05-18

**Authors:** Sascha Kuerti, Leticia Oliveira-Ferrer, Karin Milde-Langosch, Barbara Schmalfeldt, Karen Legler, Linn Woelber, Katharina Prieske, Sven Mahner, Fabian Trillsch

**Affiliations:** ^1^ Department of Gynaecology and Gynaecologic Oncology, University Medical Center Hamburg-Eppendorf, Hamburg 20246, Germany; ^2^ Department of Obstetrics and Gynaecology, University of Munich, Munich 81377, Germany

**Keywords:** ovarian cancer, (VEGF)-A, -C, -D, mesenchymal phenotype, tumour dissemination, lymph node metastases

## Abstract

**Background:**

Peritoneal dissemination and retroperitoneal lymph node involvement are main routes for progression of epithelial ovarian cancer (EOC). Vascular endothelial growth factor (VEGF) mediated angiogenesis has been identified as an important mechanism promoting tumour progression.

**Methods:**

Tumour tissue of 100 patients with EOC was analysed for protein expression of vascular endothelial growth factor (VEGF)-A, -C, -D by Western Blot analysis. Expression patterns in patients with ‘extensive intraperitoneal’ metastases (pT3c pN1 and pT3b-pT3c pN0, n=80) were compared to patients with ‘predominantly retroperitoneal’ metastases (pT1a-pT3b, pN1, n=20). Overall and progression-free survival was analysed by Kaplan-Meier method.

**Results:**

While no significant differences in expression levels among the different modes of metastases were noted for VEGF-A and -D, VEGF-C expression was significantly higher in the group of predominantly retroperitoneal metastases compared to the group with extensive intraperitoneal metastases. Patients with high VEGF-C expression had a significantly worse overall survival compared to patients with low expression levels.

**Conclusions:**

Retroperitoneal tumour progression in EOC patients is associated with high VEGF-C expression. VEGF-C may serve as a molecular marker to identify patients with assumed high risk for lymphatic metastases, who might benefit from specific treatment strategies.

## INTRODUCTION

Epithelial ovarian cancer (EOC) represents the leading cause of death in gynaecological malignancies [1]. EOC progresses mainly through extensive intraperitoneal and/or retroperitoneal (lymphatic) tumour spread, while distant metastases are rarely seen [2]. So far, the biologic background of these two different modes of progression is poorly understood. Recent investigations have shown that the tumour cell differentiation status (epithelial-mesenchymal-transition) may influence the route of metastases in ovarian cancer [3]. Accordingly, we could previously show that strong expression of an 85 kDa fragment of the epithelial marker E-Cadherin in ovarian cancer cells appears to be associated with intraperitoneal metastases [4]. Contrarily, other analyses revealed that mesenchymal tumour cells exhibited locally restricted tumour growth [3]. Highly metabolic tumour cells in locally growing tumour masses are dependent on vascular endothelial growth factor (VEGF) mediated angiogenesis [5]. The VEGF family consists of VEGF-A,-C,-D and induces different cascades via their receptor-tyrosine-kinases VEGFR-1, VEGFR-2, VEGFR-3 to exhibit their various biological effects. In this context, VEGF-C and -D bind VEGFR-3 and promote additional lymphangiogenesis [6].

The aim of the present study was to identify the diagnostic potential of the VEGF family for identification of ovarian cancer patients being at high risk for retroperitoneal metastases. Identifying these patients may enable tailored therapeutic strategies to improve their prognosis and reduce morbidity.

## RESULTS

### Tumours with lymph node metastases exhibit high levels of VEGF-C expression

Tumour samples of all included 100 patients were analysed for VEGF-A, VEGF-C and VEGF-D expression by Western Blot, including 80 (80%) with ‘extensive intraperitoneal’ tumour growth and 20 patients (20%) with ‘predominant retroperitoneal’ tumour involvement. Patient characteristics were balanced between both groups except for an expected higher rate of large bowel resections (69% vs 40% p=0.005) and lower number of resected lymph nodes (median 40 vs 49 lymph nodes p=0.043) in patients with ‘extensive intraperitoneal’ tumour growth (Table 1).

**Table 1:** Patient characteristics according to the pattern of tumour dissemination

	Overall cohort	Extensive intraperitoneal group	Predominant retroperitoneal group	p-value
**No. of patients**	100 (100%)	80 (80.0%)	20 (20.0%)	
**Age at diagnosis (years)**				0.84b
**Median**	62	62	62	
**Range**	33-84	33-84	47-75	
**Tumour stage**				**< 0.001a**
**pT1c**	3 (3%)	0 (0%)	3 (15%)	
**pT2b**	1 (1%)	0 (0%)	1 (5%)	
**pT2c**	2 (2%)	0 (0%)	2 (10%)	
**pT3a**	2 (2%)	0 (0%)	2 (10%)	
**pT3b**	13 (13%)	1 (1.25%)	12 (60%)	
**pT3c**	79 (79%)	79 (98.75%)	0 (0%)	
**Lymph node status**				**< 0.001a**
**N0**	40 (40%)	40 (50%)	0 (0%)	
**N1**	60 (60%)	40 (50%)	20 (100%)	
**Number of resected lymph nodes**				**0.043b**
**Median**	41	40	49	
**Range**	1-97	1-94	1-97	
**Mode of tumour dissemination**				**< 0.001a**
**Solely intraperitoneal group (pT2b-pT3c, pN0)**	40 (40%)	40 (50%)	0 (0%)	
**Predominant retroperitoneal group (pT1a-3b, pN1)**	20 (20%)	0 (0%)	20(100%)	
**Two-sided group (pT3c, pN1)**	40 (40%)	40 (50%)	0 (0%)	
**Grading**				0.18c
**G1**	4 (4%)	3 (3.75%)	1 (5%)	
**G2**	24 (24%)	22 (27.5%)	2 (10%)	
**G3**	72 (72%)	55 (68.75%)	17 (85%)	
**Histologic subtype**				0.09a
**Serous**	85 (85%)	71 (88.75%)	14 (70%)	
**Endometrioid**	4 (4%)	2 (2.5%)	2(10%)	
**Clear cell**	3 (3%)	1 (1.25%)	2(10%)	
**Mixed differentiation**	6 (6%)	5 (6.25%)	1 (5%)	
**Not determined/unknown**	2 (2%)	1 (1.25%)	1 (5%)	
**Surgical procedures**				
**Large bowel resection**	63 (63%)	55 (68.75%)	8 (40%)	**0.002a**
**Small bowel resection**	8 (8%)	7 (9.6%)	1 (5%)	0.35a
**Upper abdomen interventions**				
**Liver**	29 (29%)	25 (31,25%)	4 (20%)	0.23a
**Spleen**	19 (19%)	17 (21.25%)	2 (10%)	0.20a
**Pancreas**	3 (3%)	2 (2.5%)	1 (5%)	0.50a
**Postoperative residual tumour**				0.58a
**Microscopic**	78 (78%)	62 (77.1%)	16 (80%)	
**Macroscopic**	21 (21%)	17 (21.6%)	4 (20%)	
**Preoperative CA 125 level (kU/l)**				0.32b
**Median**	386	401	307	
**Range**	14-13089	14-13089	34-2802	
**Postoperative CA 125 level (kU/l)**				0.79b
**Median**	103	101	121	
**Range**	11-1267	11-1267	28-896	

Overview of the clinical characteristics of all included patients with EOC (n = 100). A total of 20 patients with predominant retroperitoneal tumour dissemination are opposed to 80 patients with extensive intraperitoneal tumour involvement. Both groups were tested with Pearson-Chi-Quadrat test (a), ANOVA analysis (b), as well as with the Kruskal Wallis (c) test upon differences. Statistically significant P values are printed in bold.

FIGO: International Federation of Gynecology and Obstetrics; kU/l: kilo Units per liter.

**Figure 1:**
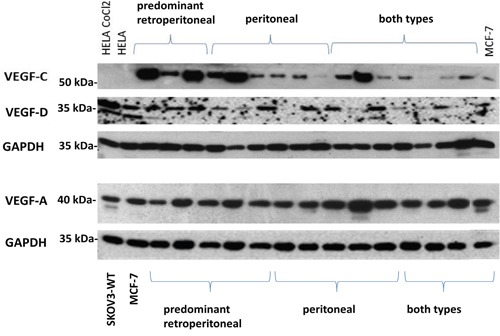
Expression of VEGF-A, VEGF-C, VEGF-D according to the mode of tumour progression Representative expression of VEGF-A, VEGF-C, VEGF-D in the different types of tumour dissemination. Protein lysates from the breast cancer cell line MCF7 were used as positive controls for VEGF-A, –C and –D. Equal amounts of protein lysate (20 μg) were loaded per well.

**Figure 2:**
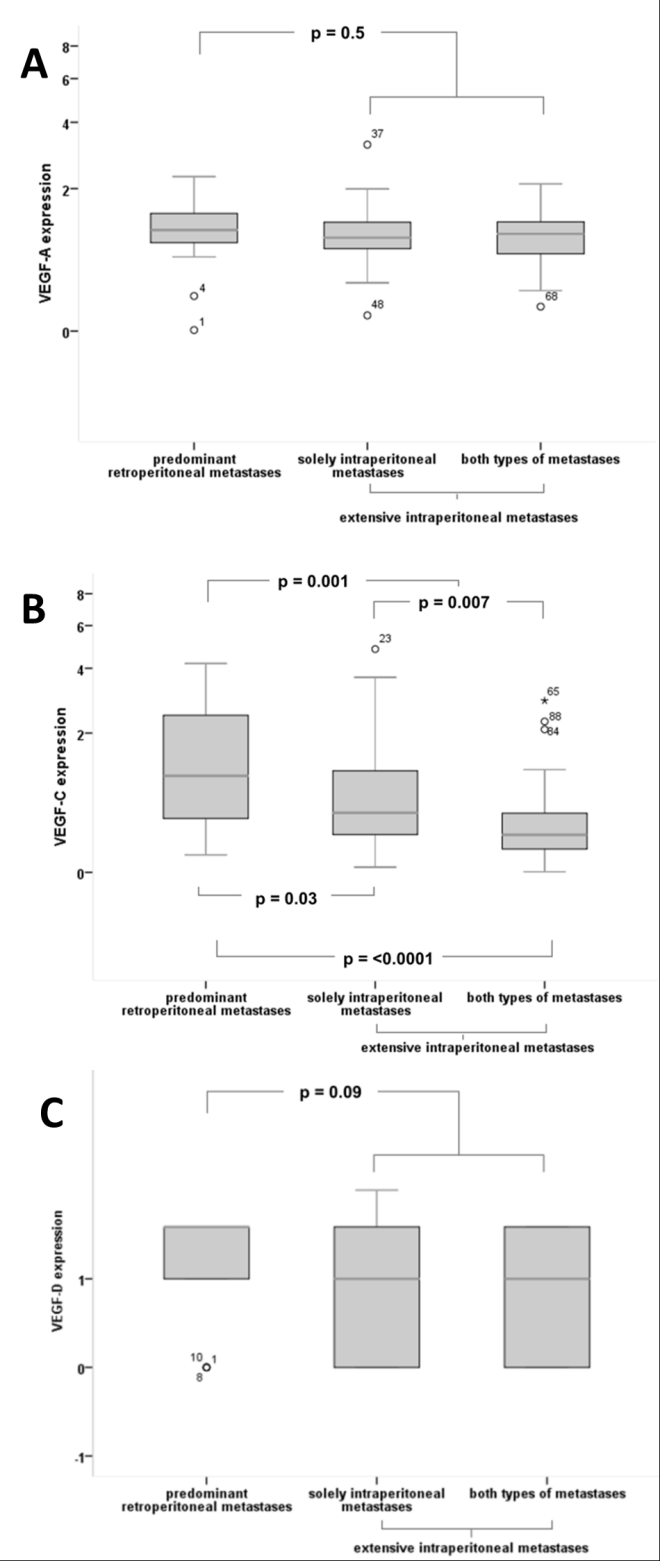
Quantitative expression of VEGF-A, VEGF-C, VEGF-D according to the mode of tumour progression **(A)** For VEGF-A no significant differences in expression levels between the different groups could be detected. **(B)** VEGF-C with significantly higher expression in patients with ‘predominant retroperitoneal‘ metastases compared to ‘extensive intraperitoneal’ metastases (median 1.14 vs 0.42, p=0.001) and compared to patients with both types of metastases (median 1.14 vs 0.35, p=0.00002), as well as compared to patients with solely intraperitoneal metastases (median 1.14 vs 0.60, p=0.03). VEGF-C expression is significantly higher in the group of solely intraperitoneal metastases compared to both types of metastases (median 0.60 vs 0.35, p=0.007) **(C)** VEGF-D exhibits a trend of higher expression levels in patients with ’predominant retroperitoneal’ metastases compared to the ’extensive intraperitoneal’ group (mean 1.33 vs 0.96, p=0.09), although without statistical significance.

For VEGF-A no significant difference in expression levels between the different groups was found (‘predominant retroperitoneal’ vs. ‘extensive intraperitoneal’ metastases: median 1.18 vs 1.09, p=0.50, Figures 1, 2A).

A significantly higher expression of VEGF-C could be detected in the ‘predominant retroperitoneal’ group compared to the ‘extensive intraperitoneal’ group (median 1.14 vs 0.42, p=0.001, Figures 1, 2B).

Expression of VEGF-C in tumours with solely intraperitoneal metastases within the ‘extensive intraperitoneal’ group significantly differed compared to tumours with retroperitoneal involvement. Median expression levels of these tumours were significantly lower compared to the ‘predominant retroperitoneal’ group (median 0.60 vs 1.14, p=0.03, Figure 2B) and higher compared to tumours with both types of metastases (median 0.60 vs 0.35, p=0.007, Figure 2B).

In accordance with VEGF-C, a semi-quantitative analysis on VEGF-D expression reveals similar trends among the different groups of metastases, although without statistical significance (‘predominant retroperitoneal’ vs extensive ‘intraperitoneal metastases’: mean 1.33 vs 0.96, p=0.09, Figures 1, 2C).

### VEGF-C correlates with postoperative residual tumour

No significant correlations between VEGF-C and VEGF-A,-D expression as well as clinical characteristics like prae-/post CA125 values, age and grading were noted. However, VEGF-C exhibits a significant correlation to postoperative residual tumour (Pearson: r=0.225, p= 0.025, Table 2).

**Table 2:** Correlation of VEGF-A,-C,-D expression and clinical/pathological patient characteristics

	VEGF-C expression	VEGF-D expression	VEGF-A expression
**VEGF-A expression**	r = 0.062; p=0.538	r = -0.004; p=0.969	
**VEGF-D expression**	r = 0.026 ; p=0.802		
**Pre-CA125 value**	r = 0.024; p=0.821	r = 0.022 ; p=0.839	r = 0.154; p=0.150
**Post-CA125 value**	r = 0.056; p=0.623	r = 0.134; p=0.256	**r = 0.242; p=0.033**
**Age**	r = - 0.058 ; p=0.569	**r = -0.205; p=0.045**	r = 0.123; p=0.223
**Grading**	r = 0.018 ; p=0.861	r = - 0.045; p=0.663	r = 0.061; p=0.544
**Postoperative residual tumour**	**r = 0.225; p=0.025**	r = 0.033; p=0.753	r = 0.0001; p=0.999

Postoperative residual tumour was categorized in ‘postoperative no tumour residual’, ‘microscopic tumour residual’, ‘tumour residual <1cm’ and ‘tumour residual > 1 cm’. p = p-value; correlation coefficient: r = Pearson. Bold values indicate statistically significant correlations.

In addition, decreasing VEGF-D values were noted with rising age of patients (Pearson: r= -0.205, p=0.045, Table 2), while VEGF-A is correlated with post-CA125 values (Pearson: r= 0.242, p=0.033, Table 2).

### Patients with high VEGF-C expression had a significantly worse overall survival

In survival analyses, no prognostic differences with regard to modes of progression were noted in terms of progression-free survival (PFS, log Rank p=0.80, Supplementary Figure 1A. and overall survival (OS), log Rank p=0.79, Supplementary Figure 1B).

Subsequently, the overall patient cohort was divided into two groups according to the median VEGF-C expression values detected by Western Blot analysis. The group of patients with low VEGF-C expression (n=50) consisted of 5 patients with ‘predominant retroperitoneal’ (10%), 17 patients with ‘solely intraperitoneal’ (34%) and 28 patients with ‘both types’ of metastases (56%). In contrast, the group with high VEGF-C expression is composed of 15 patients with ‘predominant retroperitoneal’ (30%), 23 patients with ‘solely intraperitoneal’ (46%) and 12 patients with ‘both types’ of metastases (24%, Supplementary Figure 2).

**Figure 3:**
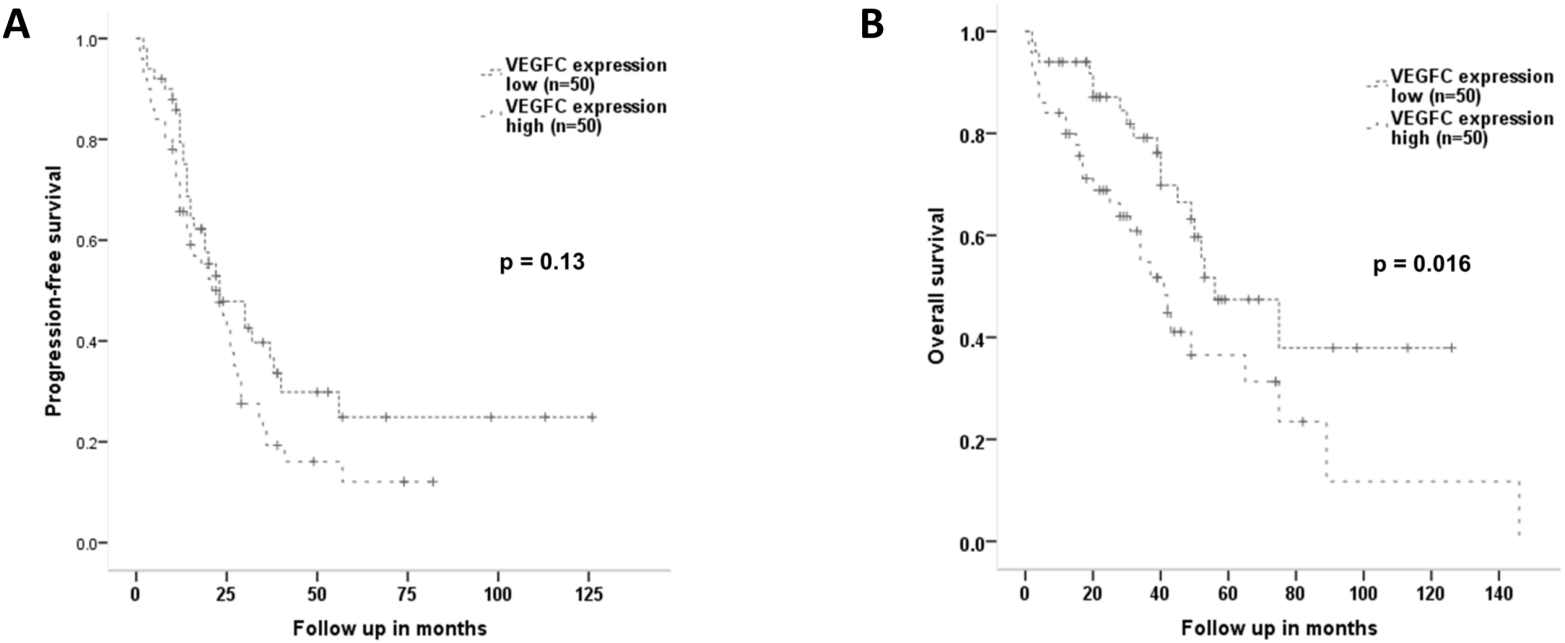
Progression-free and overall survival according to the expression level of VEGF-C By dividing the overall patient cohort into two groups according to the median VEGF-C expression by Western Blot, two groups with low (n=50) and high (n=50) VEGF-C expression were generated. **(A)** Progression-free survival shows no prognostic differences in relation to VEGF-C expression (median 23 vs 23 months; HR1.44, 95%-CI 0.89-2.31, p=0.13; Log Rank p=0.13). **(B)** Patients with high VEGF-C expression have a significantly shorter overall survival compared to patients with low VEGF-C expression levels with a median of 41 versus 56 months (HR 2.02, 95%-CI 1.12-3.63, p=0.019; Log Rank p=0.016).

Regarding PFS there was no statistically significant association with high VEGF-C expression compared to low expression (median 23 vs 23 months; HR1.44, 95%-CI 0.89-2.31, p=0.13; Log Rank p=0.13, Figure 3A). However, patients with high VEGF-C expression values had a significantly impaired OS with 41 versus 56 months (HR 2.02, 95%-CI 1.12-3.63, p=0.019; Log Rank p=0.016, Figure 3B).

## DISCUSSION

Following the results of the present explorative study including tumour samples from 100 patients with epithelial ovarian cancer, we could identify VEGF-C as a potential molecular marker attributing the risk for retroperitoneal tumour progression to ovarian cancer patients.

VEGF-C is significantly higher expressed in the group of patients with ‘predominant retroperitoneal’ metastases compared to the group of ‘extensive intraperitoneal’ tumour involvement. Although the different modes of tumour progression were not directly correlated with differences in survival, a significantly shorter overall survival for patients with high VEGF-C expression was noted, which supports a possible prognostic impact of VEGF-C mediated retroperitoneal metastases. Nevertheless, as we know that patients with extensive intraperitoneal tumor dissemination usually have an impaired prognosis, it underlines that prognostic differences are not regulated by one single factor and that multiple other factors influence the prognosis of patients. Accordingly but not-significant, VEGF-D shows similar trends among the different groups of metastases, while expression levels for VEGF-A did not differ in our cohort.

As previously described, the VEGF-C/VEGFR-3 pathway acts as an enhancer of ovarian cancer progression through autocrine and paracrine mechanisms, hence offering a potential target for therapeutic interventions [9]. Higher intratumour VEGF-C expression and worse clinical outcome of ovarian cancer patients is associated with the presence of lymphatic space invasion which may act as an indicator of lymph node metastases [10]. According to this observation, we could demonstrate that VEGF-C expression in tumours with solely intraperitoneal metastases significantly differed compared to tumours with retroperitoneal involvement.

**Figure 4:**
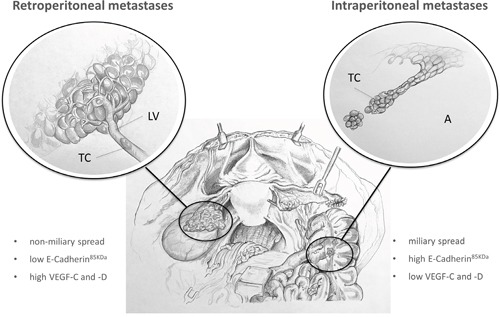
Expression patterns determining different types of metastases in EOC Tumour cells of patients with ‘predominant retroperitoneal‘ metastases are characterised by a mesenchymal tumour type with low E-Cadherin and high VEGF-C and –D expression, which probably leads to local intrapelvic tumour growth and lymphatic metastases instead of disseminating diffusely intraabdominal. The tumour type of patients with solely intraperitoneal metastases is otherwise characterised by an epithelial phenotype with high E-Cadherin and low VEGF-C and -D expression, which potentially leads to ‘extensive intraperitoneal’ tumour dissemination (A: ascites, LV: lymph-vessel, TC: tumour cell).

In line with previous investigations [3], we hypothesise that tumours with predominant retroperitoneal metastases seem to have a more mesenchymal tumour cell biology with high VEGF-C levels and low E-Cadherin expression in contrast to tumours with ‘extensive intraperitoneal’ tumour distribution [4] (Figure 4). Gene signatures in high–grade serous ovarian cancer have been identified with high VEGF-C mRNA levels in the ‘mesenchymal’ molecular subtype, which shows significantly shorter survival rates compared to ‘immunoreactive’, ‘differentiated’ or ‘proliferative’ subtypes [11]. In contrast, tumours in the subgroup with ‘solely intraperitoneal’ metastases are characterised by an epithelial phenotype with high E-Cadherin [4] as well as low VEGF-C and –D expression, which might promote intraperitoneal tumour dissemination (Figure 4). VEGF-C expression levels in tumours exhibiting intraperitoneal as well as retroperitoneal tumour involvement were lower compared to patients with solely intraperitoneal metastases which may indicate a dedifferentiation of tumour cells in tumours with both modes of progression compared to tumours with solely intraperitoneal or only predominant retroperitoneal metastases. As tumours in more advanced tumour stage with both types of metastases are characterised by a significantly lower VEGF-C expression compared to patients with predominant retroperitoneal metastases, this may indicate a potential of VEGF-C to estimate the risk for early retroperitoneal metastases. Reliable identification of patients in early stage at high risk for retroperitoneal tumour progression would be highly interesting to tailor therapeutic strategies regarding surgery and systemic therapy.

Monoclonal antiangiogenic therapy with bevacizumab, an antibody that targets VEGF-A, has been incorporated into guidelines for firstline therapy of EOC in Europe with a proven effect on progression-free survival in FIGO stages IIIB, IIIC und IV (FIGO classification before 2014, UICC 7^th^ edition) [12]. Prognostic markers and strategies for patient selection regarding antiangiogenic therapy have not been established so far. Therefore, it would be of special interest to be able to identify patients in early tumour stages, who might also benefit from additional anti-angiogenic therapy. In contrast to bevacizumab, multikinase inhibitors address several intracellular tyrosine kinases and initiate additional therapeutic effects within the antiangiogenic treatment concept [13]. In this context, cediranib serves as an example by targeting VEGFR-3 in addition to VEGFR-1, and -2. As VEGF-C and –D exert their biological effects mainly via the VEGFR-3-pathway in addition to VEGF-A, multikinase inhibitors may have the potential to be used as specific antiangiogenic drug for tumours with high VEGF-C- levels at high risk for retroperitoneal metastases. Furthermore, as a future perspective its expression might be used to characterise patients in early cancers who do not require radical systematic lymphadenectomy in staging laparotomies due to their low biological probability of lymph node metastases.

The identification of molecular markers for individual therapy is urgently required to tailor therapy of patients with EOC more individually. Even though an intraoperative decision for lymphadenectomy depending on molecular VEGF-expression is not yet possible, VEGF-C could represent a molecular marker to identify patients with high risk of lymphatic metastases who benefit from retroperitoneal surgery and specific antiangiogenic therapy (e.g. following diagnostic laparoscopy). However, further experimental and clinical investigations will be necessary to verify the benefit of an antiangiogenic therapy in form of individual VEGF-C and -D pathway inhibitors.

## MATERIALS AND METHODS

### Patients

A total of 100 patients with EOC treated at the University Medical Centre Hamburg-Eppendorf between 1995 and 2012 were included in the study. All patients underwent primary debulking surgery according to current German guidelines [7]. Patient cohorts were classified according their mode of tumour progression with two different types of metastatic spread. While a total of 20 patients had predominant retroperitoneal tumour involvement (‘predominant retroperitoneal’, pT1a-pT3b, N1), the other 80 patients exhibited extensive intraperitoneal metastases in tumour stage pT3b-pT3c, pN0 and pT3c pN1 (‘extensive intraperitoneal’). This cohort was further subdivided into a subgroup of 40 patients with extensive intraperitoneal tumour dissemination without lymph node involvement (pT3b-pT3c pN0, solely intraperitoneal metastases) and a subgroup of 40 patients with extensive intraperitoneal and retroperitoneal metastases (pT3c, N1, both types of metastases).

Detailed patient characteristics are presented in Table 1. All patients gave written informed consent to access their tissue and review their clinical records according to our investigational review board and ethics committee guidelines (#200814). Clinical data was retrieved from a detailed database providing information on clinicopathologic factors, histologic classifications and therapeutic procedures. Clinical outcome of all patients was followed from date of first diagnosis until the end of 2014.

### Tissue samples

As described previously [4] tissue samples were obtained intraoperatively and immediately stored in liquid nitrogen as fresh frozen samples. The histological characteristics of each sample were evaluated on cryo-cut and haematoxylin-eosin-stained sections. The tissue was tailored if necessary to obtain at least 70% tumour cells in the sample used for protein extraction.

### Protein extractions

As described previously [4] samples of approximately 100 mg were cut from the tissue and pulverised using a micro-dismembrator (Braun-Melsungen, Melsungen, Germany) for 2 minutes and 45 seconds at 200 r.p.m. Proteins were lysed in ice-cold sample buffer (50mM Tris pH 6.8, 1% sodium dodecyl sulphate (SDS), 10% sucrose and 10 μl/ml protease inhibitor cocktail (Sigma, Taufkirchen, Germany)). The protein concentration was determined following standard protocols and using bovine serum albumin protein standards.

### Western blot analysis

For Western blot analysis equal amounts of protein lysate (20 μg) were analysed per well. The proteins were separated on a 10 percent polyacrylamide gel (11% Glycerin, aqua dest., 10% SDS, Tris and 10% Acrylamid) and transferred to polyvinylidene difluoride membranes (Merck Millipore KGaA Darmstadt, Germany). After that, the membranes were blocked with 5% nonfat dry milk (Bio-Rad Laboratories, USA) in TBST (TBS: 50mM Tris, 150mM NaCl, pH 7.4 with 10% TWEEN 20), washed in TBST and incubated with the primary antibody. The monoclonal antibody for VEGF-A (Abcam ab46154), 1:8000, Cambridge United Kingdom) was diluted in 5% nonfat dry milk in TBST, whereas the monoclonal antibodies for VEGF-C (sc374628, 1:200, Santa Cruz Biotechnology, Heidelberg, Germany) and VEGF-D (H144 sc13085, 1:1000, santa cruz) were diluted in 5% BSA in TBST. All primary antibodies were incubated at 4°C overnight. After incubation, membranes were washed in TBST and incubated with the corresponding secondary antibody (anti rabbit sc 2054, for VEGF-A and –D; anti mouse sc 2005 for VEGF-C, all purchased from santa cruz) in 1.5% nonfat dry milk in TBST for one hour at room temperature. Finally, after washing in TBST, the detection was carried out with the SuperSignal West Pico chemoluminescent kit (Thermo Scientific, Rockford, USA) to visualise the protein expression on FUJI-super RX medical x-ray films (Tokyo, Japan).

Band intensities were quantified by densitometry (GS-700 Imaging Densitometer, BioRad, Munich, Germany). Protein lysates from the breast cancer cell line MCF7 were used as positive controls for VEGF-A, –C and -D. All detected bands of the investigated proteins except for VEGF-D were standardised with the positive controls, which were defined as 100 percent. Expression values were normalised to GAPDH (Santa Cruz FL 335; 1:5000), as loading control. VEGF-D was analysed in a semi-quantitative way because of unmanageable background signals. For VEGF-C, bands at 60 kDa were detected in our collective. As previously reported we assessed the 60 kDa band as heterodimer of the active VEGF-C monomers of 29 kDa and 31 kDa [8].

### Statistical analysis

Values representing VEGF-A, VEGF-C and VEGF-D expression levels were logarithmised and tested among the different cohorts by ANOVA analysis, as well as post hoc with the Least Significant Difference Test (LSD). VEGF-C expression was correlated by Pearson with the VEGF-A and -D as well as with clinical- and pathological parameters. P-values <0.05 were considered to be statistically significant. Boxplots were diagrammed on the basis of absolute VEGF expression levels. Univariate survival analyses were made by Kaplan Meier method and cox regression and are not independent findings. All statistical analyses were carried out with SPSS (IBM SPSS Statistics version 22 for windows).

## SUPPLEMENTARY FIGURES



## References

[1] Ferlay J, Soerjomataram I, Dikshit R, Eser S, Mathers C, Rebelo M, Parkin DM, Forman D, Bray F (2015). Cancer incidence and mortality worldwide: sources, methods and major patterns in GLOBOCAN 2012. Int J Cancer.

[2] Prat J, Oncology FCoG (2014). Staging classification for cancer of the ovary, fallopian tube, and peritoneum. Int J Gynaecol Obstet.

[3] Auer K, Bachmayr-Heyda A, Aust S, Sukhbaatar N, Reiner AT, Grimm C, Horvat R, Zeillinger R, Pils D (2015). Peritoneal tumor spread in serous ovarian cancer-epithelial mesenchymal status and outcome. Oncotarget.

[4] Trillsch F, Kuerti S, Eulenburg C, Burandt E, Woelber L, Prieske K, Eylmann K, Oliveira-Ferrer L, Milde-Langosch K, Mahner S (2016). E-Cadherin fragments as potential mediators for peritoneal metastasis in advanced epithelial ovarian cancer. Br J Cancer.

[5] Duncan TJ, Al-Attar A, Rolland P, Scott IV, Deen S, Liu DT, Spendlove I, Durrant LG (2008). Vascular endothelial growth factor expression in ovarian cancer: a model for targeted use of novel therapies?. Clin Cancer Res.

[6] Masoumi Moghaddam S, Amini A, Morris DL, Pourgholami MH (2012). Significance of vascular endothelial growth factor in growth and peritoneal dissemination of ovarian cancer. Cancer Metastasis Rev.

[7] Wagner U, Harter P, Hilpert F, Mahner S, Reuss A, du Bois A, Petru E, Meier W, Ortner P, Konig K, Lindel K, Grab D, Piso P (2013). S3-Guideline on diagnostics, therapy and follow-up of malignant ovarian tumours: short version 1.0 - AWMF registration number: 032/035OL, June 2013. Geburtshilfe und Frauenheilkunde.

[8] Joukov V, Sorsa T, Kumar V, Jeltsch M, Claesson-Welsh L, Cao Y, Saksela O, Kalkkinen N, Alitalo K (1997). Proteolytic processing regulates receptor specificity and activity of VEGF-C. EMBO J.

[9] Decio A, Taraboletti G, Patton V, Alzani R, Perego P, Fruscio R, Jurgensmeier JM, Giavazzi R, Belotti D (2014). Vascular endothelial growth factor c promotes ovarian carcinoma progression through paracrine and autocrine mechanisms. Am J Pathol.

[10] Hisamatsu T, Mabuchi S, Sasano T, Kuroda H, Takahashi R, Matsumoto Y, Kawano M, Kozasa K, Takahashi K, Sawada K, Matsuo K, Tamada Y, Morii E (2015). The significance of lymphatic space invasion and its association with vascular endothelial growth factor-C expression in ovarian cancer. Clin Exp Metastasis.

[11] Konecny GE, Wang C, Hamidi H, Winterhoff B, Kalli KR, Dering J, Ginther C, Chen HW, Dowdy S, Cliby W, Gostout B, Podratz KC, Keeney G (2014). Prognostic and therapeutic relevance of molecular subtypes in high-grade serous ovarian cancer. J Natl Cancer Inst.

[12] Heitz F, Harter P, Barinoff J, Beutel B, Kannisto P, Grabowski JP, Heitz J, Kurzeder C, du Bois A (2012). Bevacizumab in the treatment of ovarian cancer. Adv Ther.

[13] Mahner S, Woelber L, Mueller V, Witzel I, Prieske K, Grimm D, Keller VAG, Trillsch F (2015). Beyond bevacizumab: an outlook to new anti-angiogenics for the treatment of ovarian cancer. Front Oncol.

