# Supplementary material for: VEGF-C expression attributes the risk for lymphatic metastases to ovarian cancer patients
Source: Oncotarget. 2017 May 18;8(26):43218–27. doi: 10.18632/oncotarget.17978 (PMC5522140; doi:10.18632/oncotarget.17978)
Supplement: Supplementary file 1 [file oncotarget-08-43218-s001.pdf]

VEGF-C expression attributes the risk for lymphatic metastases to ovarian cancer patients

SUPPLEMENTARY MATERIALS

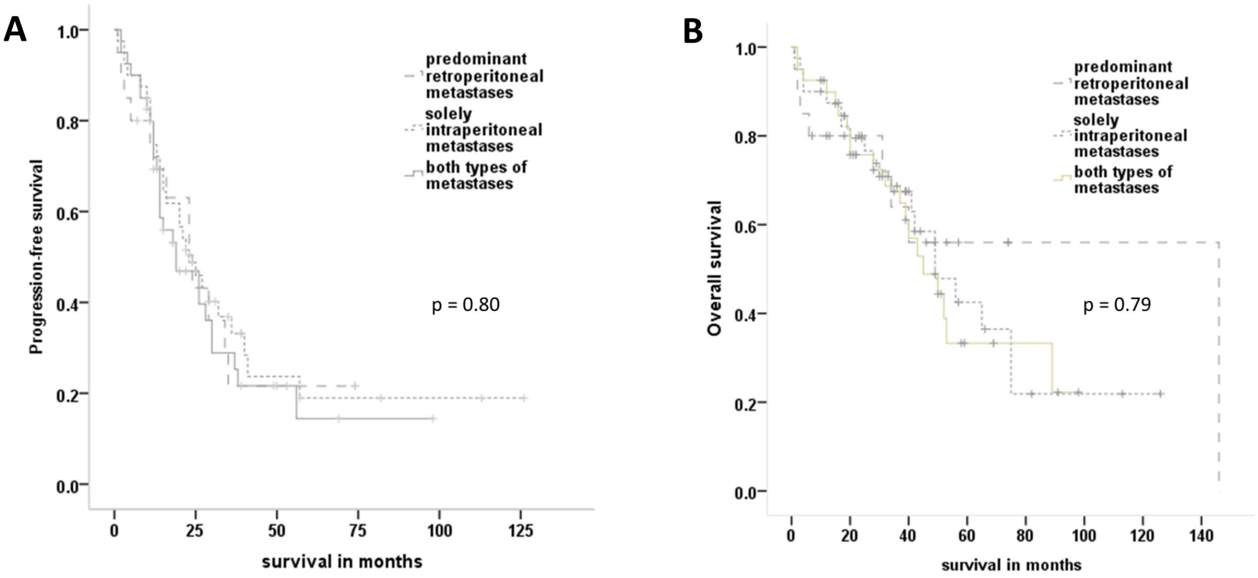

Supplementary Figure 1: Progression-free and overall survival according to type of metastases. (A) Progression-free survival shows no prognostic differences in relation to the mode of progression (median 24 (predominant retroperitoneal metastases) vs 23 (solely intraperitoneal metastases) vs. 19 (both types of metastases) months; Log Rank p=0.80). **(B)** Patients with 'predominant retroperitoneal metastases' have a longer overall survival (median 146 months) compared to patients with 'solely intraperitoneal metastases' (median 49 months) and 'both types of metastases' (median 45 months), although without statistical significance (Log Rank p=0.79).

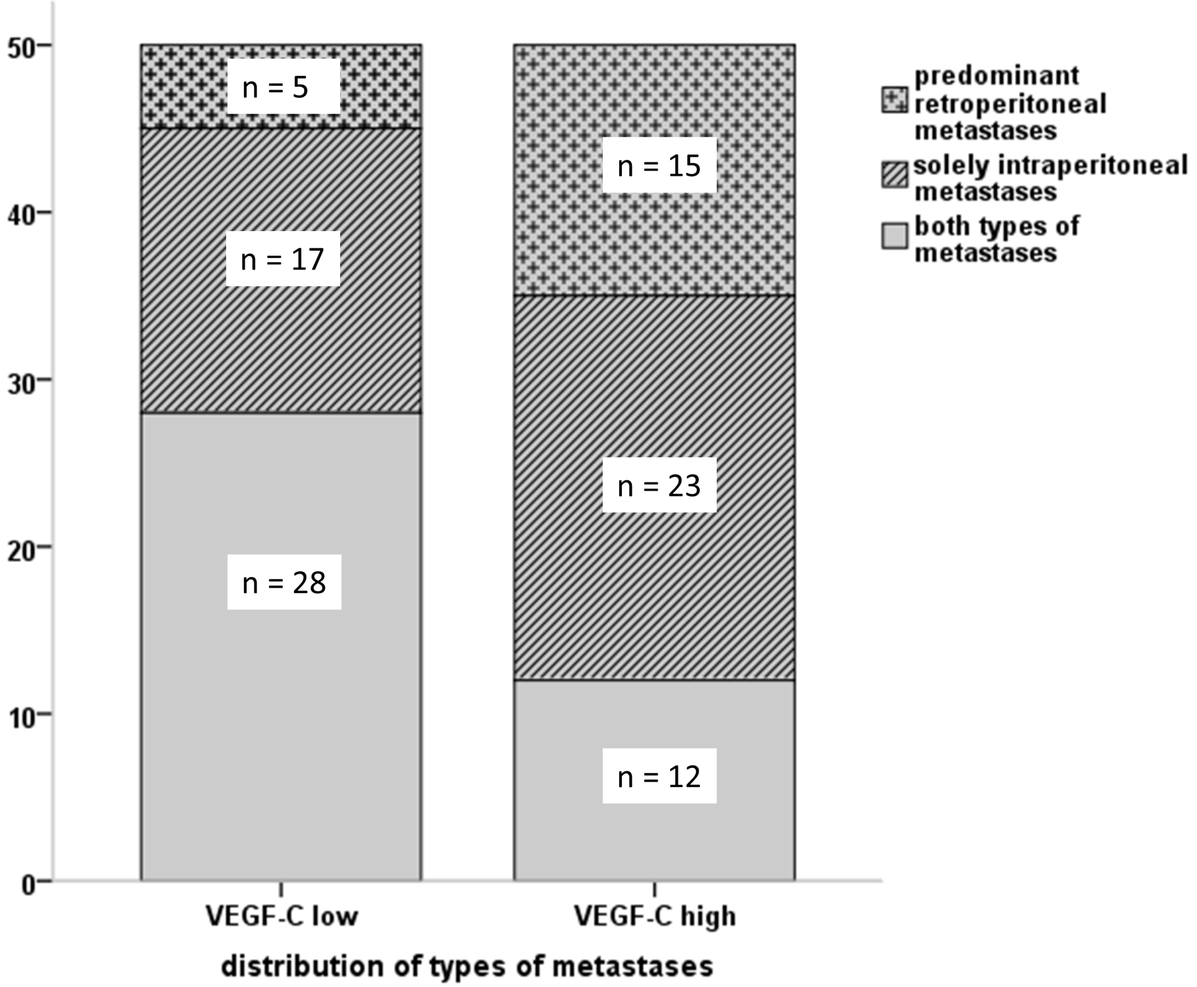

Supplementary Figure 2: Distribution of the mode of metastases in the groups of high and low VEGF-C expression. The group of patients with low VEGF-C expression (used for Kaplan Meier survival analysis) is composed of 5 patients with predominant retroperitoneal, 17 patients with solely intraperitoneal metastases and 28 patients with both types of metastases. The group of patients with high VEGF-C expression (used for Kaplan Meier survival analysis) is composed of 15 patients with predominant retroperitoneal, 23 patients with solely intraperitoneal metastases and 12 patients with both types of metastases.
